# Severe Hearing Loss in the World's First Successfully Captive‐Born Yangtze Finless Porpoise: Impact of High Underwater Sound Exposure and Congenital Hearing Disorders

**DOI:** 10.1111/1749-4877.12973

**Published:** 2025-04-06

**Authors:** Zhitao Wang, Tomonari Akamatsu, Kexiong Wang, Ding Wang

**Affiliations:** ^1^ School of Marine Science Ningbo University Ningbo Zhejiang China; ^2^ Institute of Hydrobiology Chinese Academy of Sciences Wuhan Hubei China; ^3^ Research Organization for Nano & Life Innovation Waseda University Tokyo Japan

**Keywords:** aquarium, auditory evoked potential, underwater noise, Yangtze finless porpoise

## Abstract

Aquariums globally have seen significant growth in recent decades. However, persistent negligence exists concerning underwater sound pollution in aquariums and its impact on cetaceans. Here, the audiogram of Taotao, the world's first successfully captive‐born and bred Yangtze finless porpoise, and the underwater sound levels in the Baiji Aquarium at the Institute of Hydrobiology, Chinese Academy of Sciences were examined. In contrast to the previously published U‐shaped audiograms of the Yangtze finless porpoise, Taotao's audiogram exhibited a W‐shaped pattern. Additionally, the audiogram of Taotao was, on average, 42 ± 15 dB (mean ± SD) higher than that of other non‐aquarium‐born Yangtze finless porpoises in the Baiji Aquarium and 43 ± 11 dB higher than that of wild Yangtze finless porpoises, particularly in the 20–60 kHz range and at 90 kHz. The underwater sound spectra in the Baiji Aquarium do not account for the notches observed in the porpoise's audiogram below 60 kHz, suggesting that congenital hearing disorders may be the cause of Taotao's hearing loss in these frequency bands. In contrast, the cumulative weighted broadband sound exposure levels of underwater sound within the aquarium (mean: 162 dB) exceeded the temporary threshold shift onset threshold for non‐impulsive noise (153 dB) and the permanent threshold shift onset threshold for impulsive noise (155 dB) in finless porpoises. The high levels of underwater sound exposure, particularly from conspecific porpoises, highlight the need for increased focus on the welfare of captive animals.

## Graphical abstract

The audiogram of the world's first successfully captive‐born Yangtze finless porpoise was on average 40 dB higher than conspecifics. Congenital hearing disorders and noise exposure may be the primary cause of porpoise's hearing loss.

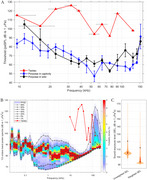

**Abbreviations**
AEP: auditory evoked potential
df: degrees of freedom
FT: Fourier transformation
peSPL: peak‐to‐peak equivalent sound pressure level
PTS: permanent threshold shift
q.d.: quartile deviation
rms: root mean square
SPLzp: zero to peak sound pressure level
SELuw: unweighted sound exposure levels
SELw: marine mammal auditory‐weighted sound exposure levels
TTS: temporary threshold shift

## 1 Introduction

Vocalization and hearing are essential for the critical life functions of cetaceans (Elemans et al. 2024; Madsen et al. 2023; Wang et al. 2022). However, underwater anthropogenic noise pollution can disrupt these crucial functions and impact odontocete echolocation abilities and well‐being (Duarte et al. 2021; Wang et al. 2020a, 2021a, 2021b). Prolonged and recurrent exposures to stressors from noise present challenges for marine mammals (Wang et al. 2020a, 2021a, 2014; Wright et al. 2007). When subjected to prolonged sound exposure, temporary threshold shifts (TTSs) (Finneran et al. 2023a, 2023b; Mooney et al. 2009; Nachtigall et al. 2016; Popov et al. 2011; Stevens et al. 2021) and permanent threshold shifts (PTSs) (Southall et al. 2019) can be induced and manifested in the audiograms of cetaceans. Intense sound exposure can even cause cetaceans to strand and, in certain circumstances, subsequently lead to death (Evans and England 2001; Schrope 2002; Simonis et al. 2020), whereas noise from underwater detonations can result in direct mortality (Danil and St. Ledger 2011).

Aquariums worldwide have undergone significant growth in recent decades (China Cetacean Alliance 2019; Tidière et al. 2023). In 2019, more than 3600 cetaceans were housed in approximately 350 publicly accessible facilities spread across 58 countries (China Cetacean Alliance 2019; Tidière et al. 2023; World Animal Protection 2019). Marine mammals housed in aquariums, as well as rehabilitation or research facilities, play a significant role in education, research, and animal conservation (Finneran et al. 2023a, 2023b; Madsen et al. 2023; Nachtigall and Supin 2013; Sørensen et al. 2023; Tidière et al. 2023).

In contrast to natural marine or freshwater ecosystems, within the domain of aquarium environments, it is theorized that detrimental acoustic stimuli may originate from the reverberations generated by the reflective properties of concrete in pool settings (Stevens et al. 2021). Moreover, the tanks are routinely outfitted with a life support pump system that generates noise characterized by elevated sound levels. Although some studies have investigated the effects of ambient sound exposure on cetaceans in captive settings (Heise et al. 2016; Houser et al. 2019; O'neal 1998; Rose et al. 2017; Scheifele et al. 2012; Starke and Scheifele 2008; Stevens et al. 2021), additional research is needed to determine the extent to which elevated ambient sound levels can impact captive cetaceans.

The Yangtze finless porpoise (*Neophocaena asiaeorientalis asiaeorientalis*) is the only freshwater porpoise in the world and presently restricted to the main stem of the Yangtze River and two adjacent lakes, namely Poyang and Dongting Lakes (Wang 2009; Wang et al. 2013). The Yangtze finless porpoise has been classified as Critically Endangered on the International Union for Conservation of Nature Red List of Threatened Species (Wang et al. 2013). The most recent survey suggests that the overall wild population comprises approximately 1249 individuals. In addition to other threats such as pollution and habitat degradation (Wang 2009; Wang et al. 2013), underwater noise has been a prominent threat (Wang et al. 2020a, 2021a, 2021b). Aquarium breeding, coupled with the potential for subsequent release into the wild, represents a crucial strategy for conserving this distinctive species and preventing its extinction (Wang et al. 2005). Thus, the auditory abilities of cetaceans born or raised in captive environments necessitate investigation.

The audiogram stands as one of the fundamental measures of animal hearing ability (Wang and Houser 2023; Wang et al. 2020b). The hearing capabilities of cetaceans can be explored through non‐invasive electrophysiological techniques, such as recording the auditory brainstem response to auditory stimuli (Popov et al. 2011, 2021; Supin et al. 2001). This electrophysiological approach has been employed in assessing the hearing capabilities of more than 30 cetacean species (Mooney et al. 2018; Mulsow et al. 2020; Sysueva et al. 2018; Wang and Houser 2023; Wang et al. 2020b, 2021c). The primary objective of this study is to examine the auditory thresholds of a Yangtze finless porpoise named Taotao by employing the auditory brainstem response method within the Baiji Aquarium, a research facility of the Institute of Hydrobiology of the Chinese Academy of Sciences. Established in 1992, the facility has been dedicated to the captive breeding and research of endangered Yangtze freshwater cetaceans. Taotao, born in July 2005, is renowned as the world's first successful captive‐born and bred Yangtze finless porpoise (Wang et al. 2005).

The secondary objective of this study is to assess the underwater sound conditions within the Baiji Aquarium, evaluate the potential impact of underwater noise exposure on Taotao's hearing, and propose potential noise mitigation methods.

## 2 Materials and Methods

### 2.1 Experimental Setting and Subject of Hearing Test

Auditory research was conducted on Taotao, housed in the main pool of the Baiji Aquarium, from March 25 to March 31, 2019. The porpoise measured a body length of 164 cm and a body weight of 47 kg. The pool is 3 m deep, 25 m wide, and 7 m long, with a kidney‐shaped configuration. The auditory assessment was conducted when the porpoise was approximately 14 years old and had reached adulthood. Notably, the Yangtze finless porpoise typically reaches maturity around 6 years of age, with a lifespan exceeding 30 years (Mei et al. 2012; Wang et al. 2013). The porpoise was trained to float on the water surface after receiving instructions conveyed by the trainer.

### 2.2 Stimuli Presentation

Electrophysiological measurements are based on the recording of auditory evoked potentials (AEPs) (Finneran 2015; Wang et al. 2025), similar to those used in electroencephalogram (EEG) measurements in humans (Supin et al. 2001). Given the observed heightened rate of following responses to pulse stimuli featuring modulation rates of 1 kHz within the auditory systems of numerous toothed whales (Smith et al. 2018), as well as the finless porpoise (Mooney et al. 2011), the presentation rate of tone pip stimuli in this study was configured to 1 kHz. The AEP test utilized rhythmic tone pips arranged in sequences, with the pip train stimulus administered at a rate of 10 cycles per second (Wang et al. 2020b, 2021c). Each stimulus, comprising a series of pips, consisted of 20 individual pips, each lasting 0.25 ms and subjected to cosine‐envelope modulation (Figure S1). For each distinct stimulus test, the carrier frequencies of the pip train were adjusted, and a sequence of 10 carrier frequencies (9.5, 16.0, 22.6, 32.0, 38.0, 53.9, 64.0, 76.1, 90.5, 128.0 kHz) was analyzed. Stimulus signals were generated utilizing a bespoke LabVIEW program (National Instruments, Austin, TX, USA) on a laptop computer. Subsequently, these signals underwent digital‐to‐analog conversion via an NI multifunctional I/O card (NI USB‐6251 BNC) operating at an update rate of 512 kHz. The analog data underwent successive attenuation and amplification stages utilizing an HP attenuator (HP‐350D, Hewlett‐Packard, Palo Alto, CA, USA) and an HP power amplifier (HP‐465A), respectively. Finally, the attenuated signals were transmitted to a Reson transducer (TC‐4040, Teledyne Reson, Slangerup, Denmark) for broadcasting. The TC4040 offers a practical frequency range spanning from 1 Hz to 130 kHz, alongside a transmitting sensitivity of 132 dB re 1µPa/V at 1 m (at 50 kHz). In odontocete cetaceans, sound stimuli traverse the animal's acoustic window and propagate to the inner ear (Norris 1968). In this study, the acoustic center of the transducer was positioned 1 m from the whale's acoustic window and at a depth of 0.3 m below the water surface. To evaluate the stimuli, signals were monitored in real‐time using a Tektronix oscilloscope (TDS1002C, Beaverton, OR, USA) before transmission to the transducer.

### 2.3 AEP Measurements

The AEP was recorded using three suction cup electrodes, each featuring a 10‐mm gold‐plated Grass electrode (Model: F‐E5GH, Manufacturer: Grass Technologies, Astro‐Med., West Warwick, RI, USA). Throughout the data collection procedure, one electrode was situated approximately 6–8 cm posterior to the blowhole along the midline of the odontocete's dorsal surface, precisely positioned over the caudal aspect of the brain case, functioning as the recording electrode. A reference electrode was securely placed roughly 40 cm posterior to the recording electrode along the dorsal midline. Additionally, a ground electrode was affixed to the tail of the animal. Before attaching the electrodes to the animal, a highly conductive Signa gel electrolyte (Signagel, Parker Laboratories, Inc., Fairfield, NJ, USA) was applied to the tip of each electrode. AEP responses were acquired via electrodes and amplified 20 000 times using a Grass bioelectrical amplifier (Grass CP511; Astro‐Med, Inc., West Warwick, RI, USA). The amplified signals underwent bandpass filtering (300‐3000 Hz) using a CP511 bioelectrical amplifier. Subsequently, the analog AEP signals were digitized using an NI USB‐6251‐BNC multifunctional I/O card with a sampling rate of 16 kHz and a recording length of 30 ms. A coherent averaging procedure, encompassing 500 individual AEP signals, was employed prior to data storage.

### 2.4 Auditory Threshold Calculation

The sound pressure level of the stimulus for each test carrier frequency was initially presented at 120 dB re 1 µPa. Subsequent adjustments to the stimulus level were made based on the presence or absence of neurological responses in the AEP. The follow‐up stimuli were either attenuated or amplified in decibel step sizes of ≤ 10 dB. An objective statistical procedure was used to determine the presence or absence of the auditory steady‐state response (ASSR), also known as the envelope following response, at each stimulus sound pressure level using the *F* ratio (Finneran 2015). In the frequency domain of the AEP, the power estimated at the fundamental frequency and neighboring frequencies conformed to a chi‐square distribution (Zurek 1992). The presence or absence of AEPs was assessed using the F statistical test. This objective response detection technique, based on the signal's frequency domain, determines whether the power at a specific frequency is statistically distinguishable from the noise power averaged over adjacent frequencies (Dobie and Wilson 1996). The *F*‐statistic test can be represented by the following equation:

(1) $$\begin{array}{ccc} F\;\left(f_{p}\right) & = & \frac{S\left(k1\right)}{\sum \frac{S\left(k2\right)}{m}}\;\left[k1=\frac{f_{p}}{\Delta f},k2\in \left(k1-\frac{m}{2},k1+\frac{m}{2}\right),\right.\\ & & \left.\;k2\neq k1,\Delta f=\frac{fs}{n\mathrm{fft}}\right] \end{array}$$

where *f*_p_ denotes the frequency with the maximum signal power spectrum, *F*(*f*_p_) represents the *F*‐statistic at a frequency of interest in *f*_p_, *S*(*k*1) and *S*(*k*2) denotes the *k*1−th and *k*2−th data point of the power spectrum of the evoked potential signal, respectively. The *k*1−th data point corresponds to the peak power level, Δ*f* represents the frequency resolution of the spectrum of the evoked potential signal, *f*s denotes the sample frequency of the AEP signal, *n*fft represents the number of data points in the Fourier transformation (FT) of the AEP signal, and *m* denotes the number of neighboring frequency points utilized in the *F*‐statistic test. The average noise power across *m* adjacent frequencies conforms to a chi‐square distribution with degrees of freedom (df) equal to 2 × *m* (Dobie and Wilson 1996). During the process of estimating auditory thresholds, a 16 ms segment of the signal, beginning at 5 ms relative to the onset of sound recording, was extracted to encompass the majority of AEP responses. Fourier transformation was subsequently applied to the signal slice to obtain its frequency spectra. The power spectrum of the AEP for pip stimuli presented at a 1‐kHz rate peaks at 1 kHz. In the current investigation, the sampling frequency was configured to 16 kHz, and the Fourier transformation employed 256 data points, thus yielding a frequency resolution of 62.5 Hz. The noise power was averaged across 16 adjacent frequencies (*m* = 16) centered around 1 kHz. For pip stimuli presented at a frequency of 1 kHz, the *F* ratio was computed by dividing the power spectrum at 1 kHz by the power averaged over 16 neighboring frequencies centered around 1 kHz within the frequency range from 0.5 to 1.5 kHz. The statistical significance of the *F*‐ratio was evaluated by comparing the critical value (*F*_crit_) from the standard table, with degrees of freedom set at 2 and 32, and an alpha level of 0.05 and 0.01, respectively (Zar 1999). Auditory threshold calculations were performed utilizing a bespoke acoustic analysis algorithm implemented in MATLAB R2018B (The MathWorks, Natick, MA, USA).

### 2.5 Establishing Standardized Calibration Table

The stimuli administered to the dolphin through its acoustic window were calibrated following each auditory session. Calibration procedures paralleled those utilized during AEP assessments, encompassing consistent methods for stimuli presentation and sound recording, with the sole exception being the absence of the dolphin during the calibration process. During the calibration process, a calibrated Reson hydrophone (Model: TC4013) was positioned at the designated animal location (0.3 m water depth and 1 m from the transducer). The TC4013 provides a functional frequency range spanning from 1 Hz to 170 kHz, coupled with a sensitivity level of −210 dB re 1 V/µPa. The signal received by the Reson TC4013 underwent conditioning through a Reson preamplifier (Model: VP2000, Manufacturer: Teledyne Reson, Slangerup, Denmark), featuring 50 dB amplification and bandpass filtering ranging from 50 Hz to 250 kHz. Subsequently, the conditioned signals were digitized using the multifunctional NI USB‐6251‐BNC I/O card and stored on a laptop computer. Signals recorded by the laptop computer were then measured and utilized to establish precise sound pressure levels received by the porpoise for each projected stimulus level, serving as standard calibration tables.

### 2.6 Construction of the Audiograms

The peak‐to‐peak equivalent sound pressure levels (peSPL, dB re 1 µPa) were computed for each sound stimulus. PeSPL was calculated by subtracting 9 dB from the peak‐to‐peak sound pressure level (American National Standards Institute 2018). The auditory thresholds for each carrier frequency were calibrated to their precise sound pressure levels utilizing the standard calibration table established during the calibration of stimulus signals. Audiograms were subsequently generated by plotting each stimulus carrier frequency against its corresponding hearing threshold in peSPL.

### 2.7 Ambient Noise Measurements

Noise recordings were conducted preceding the administration of the porpoise hearing test, spanning from March 5 to March 12, 2019. Ambient noise levels were measured using SoundTrap compact recorders (ST300 HF, Ocean Instruments NZ, Warkworth, Auckland, New Zealand) at a sampling rate of 576 kHz. Noise recordings were conducted continuously throughout the recording period without employing a duty cycle. The SoundTrap provides an operational frequency range ranging from 20 Hz to 150 kHz ± 3 dB, featuring an end‐to‐end calibration sensitivity at a high gain setting of 188.3 dB re 1 V/µPa. Given that the mean apparent peak‐to‐peak source level of the Yangtze finless porpoise is 167 ± 8 dB (mean ± standard deviation), with a range from 152 to 180 dB (Fang et al. 2015), and that the SoundTrap can record porpoise clicks without distortion, this equipment is well‐suited for accurately capturing these acoustic signals. The SoundTrap recorder was centrally positioned and situated at a mid‐depth level within the pool, approximately 1.5 m above the pool bottom. Acoustic parameters related to noise, comprising zero to peak sound pressure level (SPLzp, dB re 1µPa), unweighted and marine mammal auditory‐weighted sound exposure levels (SELuw and SELw, in 1 µPa^2^s), as well as 1/3 octave‐band power spectra, were calculated for each 1‐s temporal frame using the following equations (Southall et al. 2019; Wang et al. 2020a, 2021a).

(2) $$\mathrm{SP}L_{\mathrm{zp}}=20\times \log_{10}\left|p_{i}\right|$$

(3) $$\mathrm{SE}L_{\mathrm{uw}}=10\times \log_{10}\int_{\frac{fs}{M}}^{\frac{M+1}{2}\times \frac{fs}{M}}\mathrm{PSD}\left(f\right)\;df$$

(4) $$\mathrm{SE}L_{w}=10\times \log_{10}\int_{\frac{fs}{M}}^{\frac{M+1}{2}\times \frac{fs}{M}}\left[\mathrm{PSD}\left(f\right)\times 10^{\frac{W\left(f\right)}{10}}\right]df$$

(5) $$\mathrm{PSD}\;\left(f\right)=2\times {\left(\frac{\mathrm{fft}\left(p_{i},M\right)}{N}\right)}^{2},f\in \left[\frac{fs}{M},\frac{M+1}{2}\times \frac{fs}{M}\right],i\in \left[1,N\right]$$

(6) $$\begin{array}{ccc} W\;\left(f\right) & = & 1.36+10\times \log_{10}\\ & & \;\left(\frac{{\left(f\times 10^{-3}/12\right)}^{3.6}}{{\left[1+{\left(f\times 10^{-3}/12\right)}^{2}\right]}^{1.8}\times {\left[1+{\left(f\times 10^{-3}/140\right)}^{2}\right]}^{2}}\right) \end{array}$$

where *p*_i_ represents the digitized instantaneous acoustic pressure, *M* denotes the fast Fourier transform (FFT) size, *f*_s_ indicates the sample frequency of the signal, PSD(*f*) signifies the power spectral density at a specific frequency (ƒ, in Hz), fft represents the FFT function, *N* denotes the total number of points used in FFT calculations, and *W*(ƒ) denotes the attenuation amplitude (in dB) based on the marine mammal auditory weighting function at a particular frequency (ƒ, in Hz) for the very high‐frequency cetacean functional hearing group. Power spectral densities were computed with a temporal resolution of 1 s and a frequency resolution of 1 Hz, employing a 0% sample overlap between adjacent frames. Concerning the computation of 1/3 octave‐band power spectra, the PSDs were partitioned and combined following the 1/3 octave‐band standards, with the boundaries for each band derived from ANSI S 1.6‐1986. Unweighted and weighted cumulative sound exposure levels (SELcum) were calculated using the following equations:

(7) $$\text{Unweighted}\;\text{SELcum}=\text{mean(SELuw})+10\;\mathrm{lo}g_{10}\;\left(T\right)$$

(8) $$\text{Weighted}\;\text{SELcum}=\text{mean(SELw})+10\;\mathrm{lo}g_{10}\;\left(T\right)$$

where *T* is the exposure duration (in s) with a recommended accumulation period of 24 h. The noise was analyzed using a customized protocol developed in MATLAB R2018B.

## 3 Results

### 3.1 Audiogram

**FIGURE 1:**
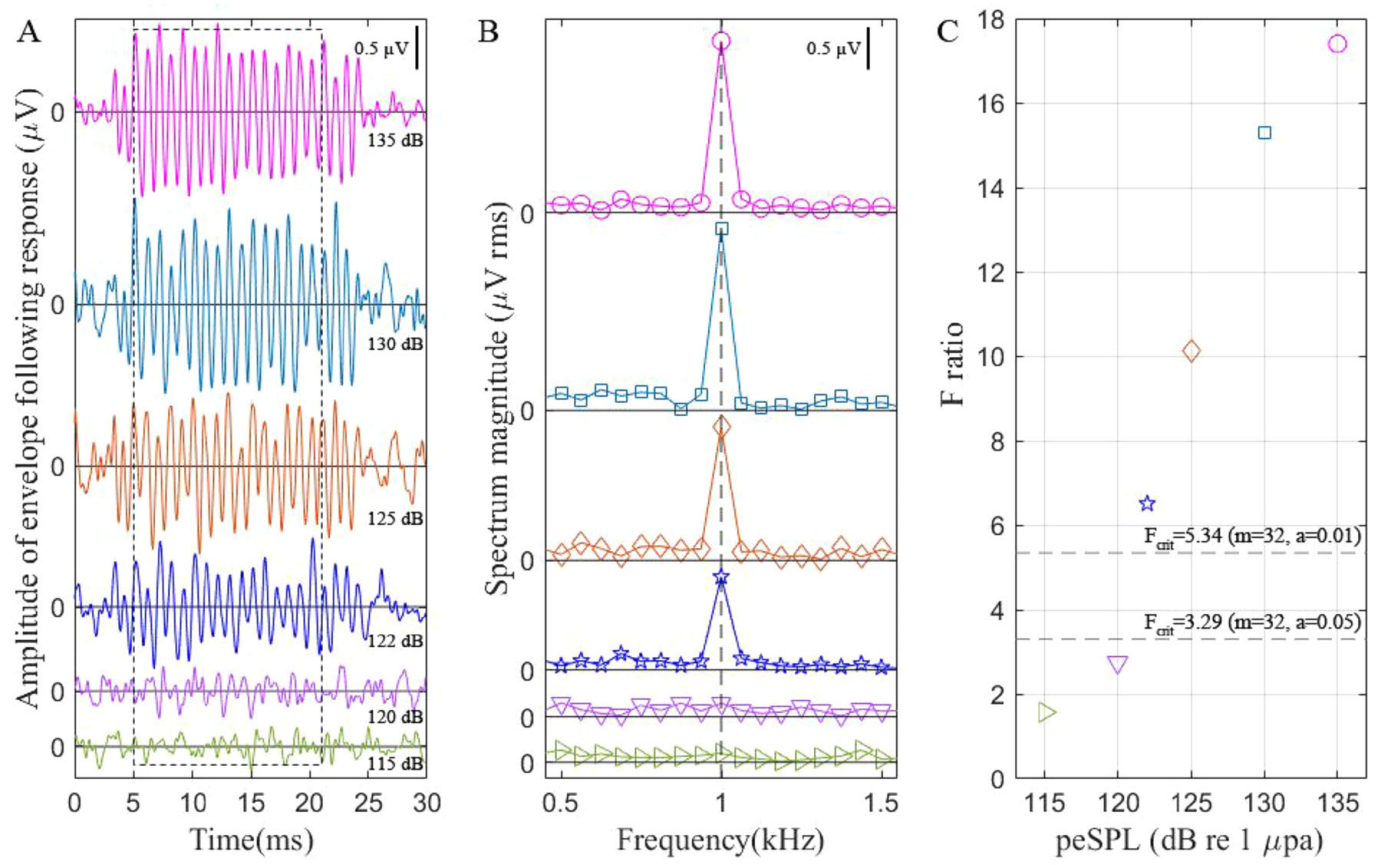
(A) Waveforms illustrating the recorded auditory evoked potential responses to sound stimuli at 38.0 kHz carrier frequencies across various peak‐to‐peak equivalent sound pressure levels (peSPL, dB re 1 µPa) triggered by the onset of pip train stimuli. The peSPL for each test is indicated in the lower right corner of the waveform. (B) The spectrum of the 16‐ms signal slice contains the predominant auditory evoked potential response. The signal slice begins at the 5‐ms time point, as depicted in (A) within the dashed box. The spectrum in B was color‐matched with its waveform in (A). (C) The *F* ratio was computed for auditory evoked potential responses as a function of peSPL. *F* ratio was computed by dividing the power spectrum at 1 kHz by the power averaged over 16 neighboring frequencies centered on 1 kHz within the frequency range from 0.5 to 1.5 kHz. The dashed line denotes the critical *F* ratio at significance levels of 0.01 and 0.05, respectively, for degrees of freedom (df) of 2 and 23.

A time lag of approximately 3 ms was observed in the AEP responses corresponding to the onset of pip train stimuli (Figure 1A). The observed time delay was attributed to the combined effects of the time taken by the stimuli to travel the 1 m distance from the stimulus projector to the animal's acoustic window and the neural processing time required for the animal to respond to the stimuli (Supin et al. 2001). The flowchart for estimating auditory thresholds is shown in Figure 1. An *F* ratio higher than 3.29 and 5.34 indicates that the corresponding peSPL for the specific stimulus frequency can elicit a significant hearing response at the *p* < 0.05 and *p* < 0.01 levels, respectively (df = 2, 32). As depicted in Figure 1, the estimated threshold for the stimulus frequency of 38.0 kHz was determined to be 122 dB re 1µPa.

**FIGURE 2:**
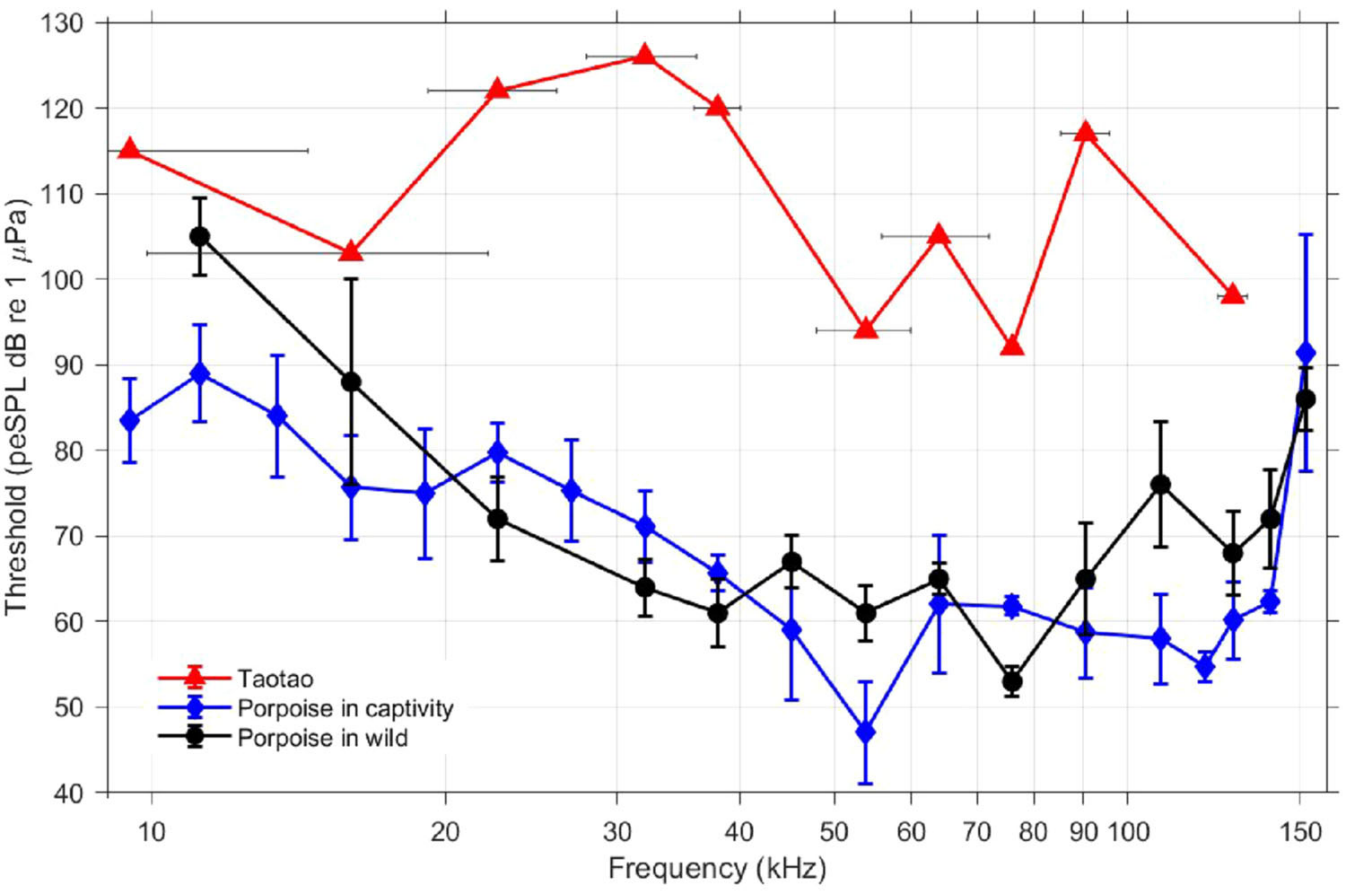
Audiograms of the Yangtze finless porpoise. The audiogram of the Yangtze finless porpoise in captivity was derived from Popov et al. (2005, 2011), while the audiogram of the Yangtze finless porpoise in the wild was derived from Wang et al. (2020b). Vertical error bars represent standard deviations from mean thresholds, while the horizontal error bar illustrates the −3‐dB bandwidth of the test stimuli.

Unlike the previously published audiogram of the Yangtze finless porpoise, the audiogram of Taotao exhibited a W‐shaped pattern (Figure 2). The most sensitive auditory frequency, marked by the lowest hearing threshold, was 76.1 kHz, reminiscent of those observed in the wild finless porpoise (Wang et al. 2020b). However, the hearing threshold (92 dB) was 39 dB higher than that observed in the wild finless porpoise (53 dB) (Figure 2). The second most sensitive auditory frequency of Taotao was 53.9 kHz, aligning with observations in the captive finless porpoise (Popov et al. 2005, 2011), where the most sensitive auditory frequency was also observed at 53.9 kHz. However, the hearing threshold (94 dB re 1µPa) was 47 dB higher than that recorded in the aquarium finless porpoise (47 dB) (Figure 2). The audiogram of Taotao was, on average, 42 ± 15 dB (mean ± SD) higher than that of the captive finless porpoise (Popov et al. 2005, 2011), and 43 ± 11 dB higher than that of the wild finless porpoise (Wang et al. 2020b).

### 3.2 Noise Level

**FIGURE 3:**
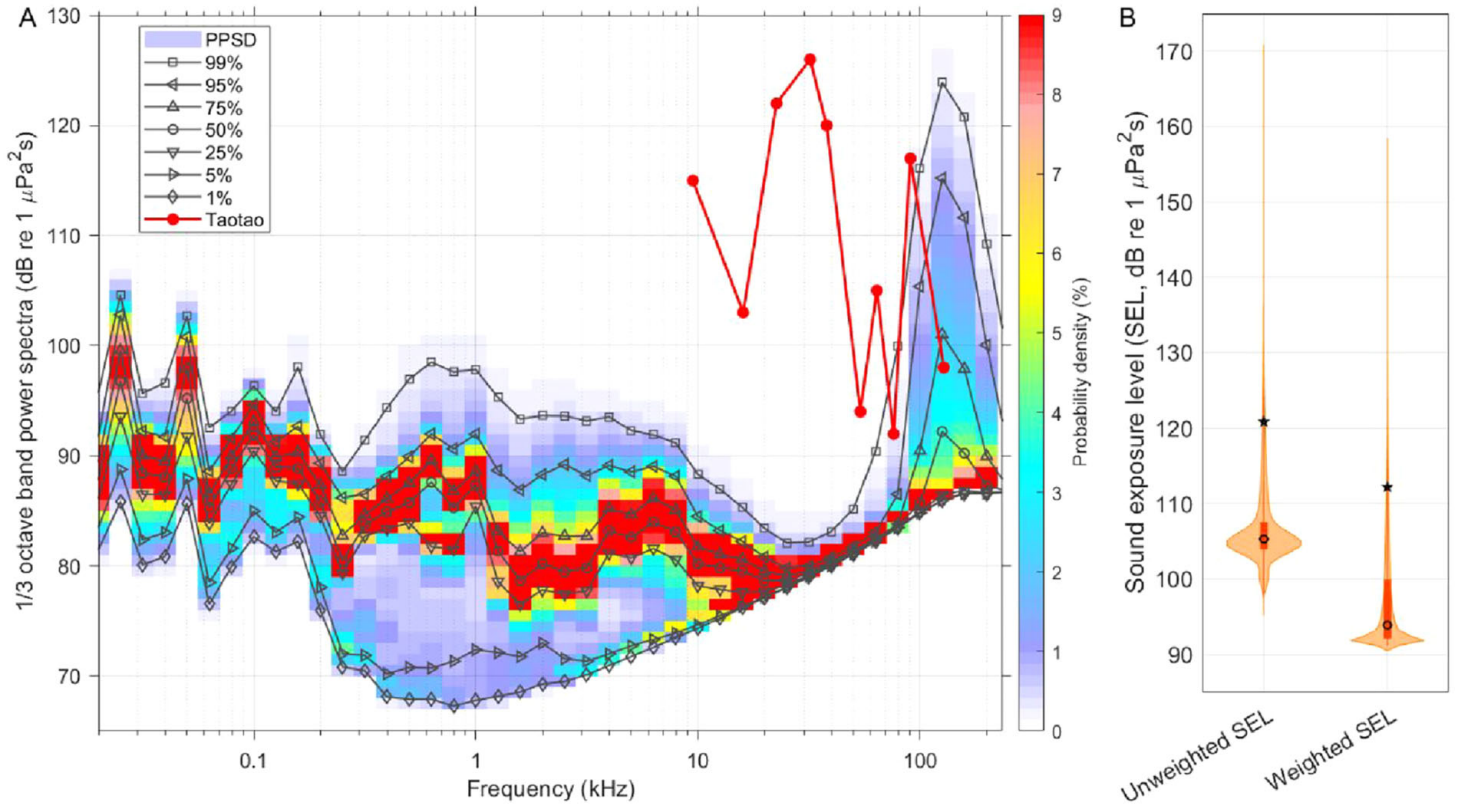
(A) Probability of power spectrum density (PPSD) of the 1/3 octave band sound pressure level of the noise in the Baiji Aquarium of the Institute of Hydrobiology, Chinese Academy of Sciences. The percentile results of 1%, 5%, 25%, 50%, 75%, 95%, and 99% of the data in an ordered set for each 1/3 octave band were also provided. The audiogram of Taotao was overlaid. (B) Violin plot depicting the unweighted sound exposure level (Unweighted SEL) and marine mammal auditory‐weighted sound exposure levels (Weighted SEL) of the noise in the Baiji Aquarium. The density of the points at a given sound exposure level is represented by the width of the plot. The black circle in each box indicates the median, while the black star represents the mean. The edges of the boxes correspond to the first and SDthird quartiles.

The noise within the Baiji Aquarium of the Institute of Hydrobiology, Chinese Academy of Sciences is concentrated within the frequency range below 10 kHz and above 90 kHz (Figure 3). The noise below 10 kHz peaked at the 1/3 bands centered at the frequencies of 25 Hz, 50 Hz, 100 Hz, 158.5 Hz, 631 Hz, and 1 kHz (Figure 3). The noise above 90 kHz peaked at the 1/3 octave band centered at the frequency of 125.9 kHz (Figure 3).

The SELuw and SELw of the noise within the Baiji Aquarium were 121 ± 7.3 dB (mean ± SD) and 112 ± 5.3 dB, respectively (Table 1). The cumulative weighted sound exposure levels of the noise within the Baiji Aquarium (mean: 162 dB) surpassed both the TTS onset threshold for impulsive noise (140 dB) and that for non‐impulsive noise (153 dB) in finless porpoise (Table 1). The cumulative weighted sound exposure level (mean: 162 dB) exceeds the established threshold for the onset of PTS caused by impulsive noise, currently set at 155 dB, but remains below the threshold for PTS onset due to non‐impulsive noise, currently set at 173 dB in finless porpoise (Table 1).

**TABLE 1:** Unweighted and weighted sound exposure levels (SEL), along with the cumulative SEL (SELcum), of the noise within the aquarium. SEL was in dB referenced to 1 µPa^2^s. qd, the quartile deviation; sd, the standard deviation. *N* represents the total sample size of noise, each with a duration of 1 s. The numeric thresholds for both temporary threshold shift (TTS) and permanent threshold shift (PTS) onset in weighted SELcum originated from Southall et al. (2019) for very high‐frequency cetaceans.

	SEL					SELcum	TTS onset threshold		PTS onset threshold	
	Median	q.d.	Mean	SD	*N*	Mean	Impulsive noise	Non‐impulsive noise	Impulsive noise	Non‐impulsive noise
Weighted	94	3.9	112	5.3	604 800	162	140	153	155	173
Unweighted	105	1.8	121	7.3	604 800	170				

**FIGURE 4:**
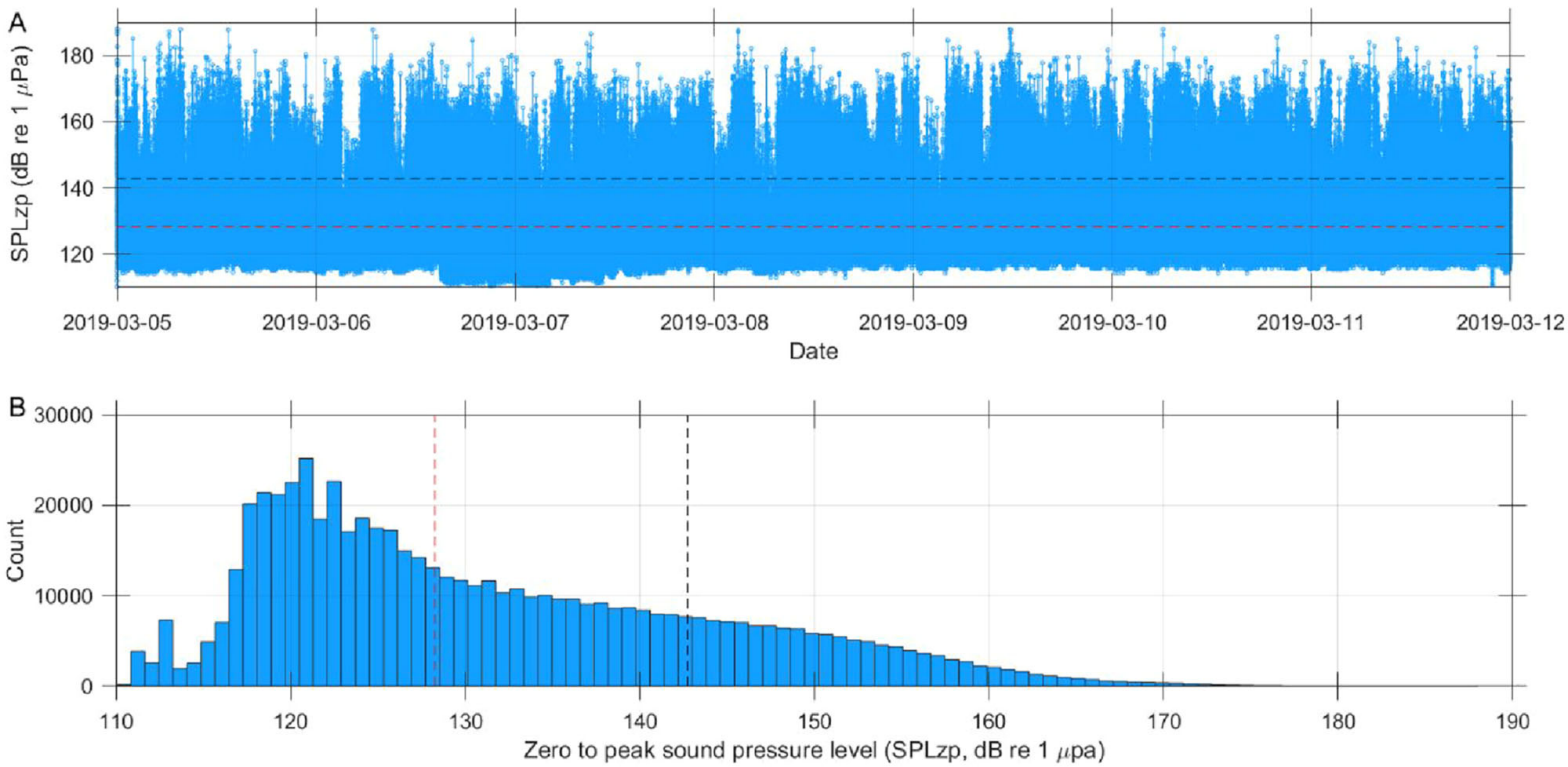
(A) Line plot and (B) histogram illustrating the zero‐to‐peak sound pressure level (SPLzp) of the noise in the Baiji Aquarium. The dashed red and black line represents the median and mean level of the SPLzp at 128.3 dB and 142.7 dB, respectively.

The mean and median zero‐to‐peak sound pressure levels (SPLzp) were 142.7 and 128.3 dB, respectively (Figure 4). Additionally, 20.85% of the noise was louder than the mean SPLzp, while 1.88% of the noise was louder than the mean SPLzp of the echolocation signal emitted by the Yangtze finless porpoise, which is estimated to be 161 dB. This estimation is derived from the peak‐to‐peak apparent source level of 167 dB re 1 µPa for clicks emitted by the finless porpoise at the Baiji Aquarium (Fang et al. 2015). Due to the high directionality of high‐frequency biosonar, it is unknown if the recorded click was from the on‐axis direction; hence, the apparent source level was used.

## 4 Discussion

### 4.1 Audiogram of Odontocetes

Aural perception serves as the principal sensory modality for odontocetes, likely shaped by evolutionary pressures inherent to their aquatic habitat. Notably, in harbor porpoises (*Phocoena phocoena*), auditory acuity reaches full maturity within a day of birth (Wahlberg et al. 2017). Odontocete audiograms exhibit the characteristic U‐shaped pattern common to mammalian auditory systems, showcasing peak sensitivity at ultrasonic frequencies (Mooney et al. 2018; Wang et al. 2020b, 2021c). Distinct from the previously documented audiograms of the Yangtze finless porpoise, Taotao's audiogram displayed a W‐shaped profile. However, Taotao's audiogram resembled that of other Yangtze finless porpoises in the peak sensitivities, albeit with higher thresholds. Specifically, the auditory frequency with the highest sensitivity in Taotao is reminiscent of those documented in wild Yangtze finless porpoises (at 76.1 kHz) (Wang et al. 2020b), while the second most sensitive frequency bears resemblance to observations in captive porpoises (at 53.9 kHz) (Popov et al. 2005, 2011). Moreover, Taotao's audiogram displayed an exceptionally elevated hearing threshold between the frequencies of 22.6 and 38 kHz. Generally, threshold shifts occur within the octave above the fatiguing sound, such that an abrupt threshold shift of more than 47 dB and a residual threshold shift of 11 dB were observed at one‐half octave (5.8 kHz) and one octave (8.2 kHz) above the exposure frequency of 4.1 kHz, respectively, in the underwater hearing sensitivity of a trained harbor seal (*Phoca vitulina*) (Reichmuth et al. 2019b). Considering that the noise power spectral density in the octave below 22–38 kHz, specifically from 10 to 20 kHz, was low; the threshold shift observed in the seal was not applicable to Taotao. The underlying cause of Taotao's markedly diminished auditory sensitivity in the 22.6 to 38 kHz frequency range remains undetermined, and congenital hearing disorders may be a primary cause of Taotao's hearing loss in these frequency bands.

Ototoxic drug treatment has also been identified as a cause of hearing loss in marine mammals in aquariums. Aminoglycoside antibiotics, such as gentamicin and amikacin, are recognized for inducing cochlear hair cell death in mammals (Matz 1986) and have been implicated as a potential cause of hearing loss in captive marine mammals (Houser and Finneran 2006). However, Taotao did not receive any ototoxic drug treatment, so drug‐induced hearing loss can be ruled out (Wang C.Q., personal communication).

The occurrence of age‐related hearing loss in marine mammals has been previously documented (Houser et al. 2008; Ridgway and Carder 1997). The audiogram of the Yangtze finless porpoise at the Baiji Aquarium was previously obtained from an 8‐year‐old male (named A Fu) and a 5‐year‐old female (named Jingjing, the mother of Taotao) in 2004 (Popov et al. 2005), and from an 11‐year‐old male (named A Bao) in 2010 (Popov et al. 2011). All previously obtained audiograms demonstrated a normal U‐shaped pattern. The relatively younger age of the finless porpoises in the former investigation, compared to the current study (with Taotao being 14 years old), may partially explain the higher hearing thresholds observed in this study, suggesting that partial presbycusis may be present in Taotao.

Additionally, extended time housed in the aquarium may also affect Taotao's hearing capacity. A Fu and Jingjing were wild‐caught animals from the Jiayu region of the Yangtze River and were transferred to the Baiji Aquarium in 1996, where they had been for nearly 8 years by the time of the auditory test in 2004 (Popov et al. 2005). The porpoise A Bao was a wild‐caught specimen from the Tianezhou Oxbow, transferred to the Baiji Aquarium in 2004, and had been there for nearly 6 years by the time of the auditory test in 2010 (Popov et al. 2011), whereas Taotao was raised in the Baiji Aquarium for 14 years at the time of hearing test in this study.

Regarding the cause of Taotao's impaired hearing, we speculate on the following possibilities. First, Taotao's hearing sensitivity at the majority of frequencies was likely damaged at birth due to congenital hearing disorders, although, to the authors' knowledge, no documented cases of congenital hearing loss in cetaceans have been reported previously. Second, it is possible that Taotao had a normal audiogram at birth, but exposure to an unknown sound source at Baiji Aquarium over the past 14 years, particularly after 2010, may have impaired its hearing sensitivity. The audiograms of the housed Yangtze finless porpoises in the Baiji Aquarium were obtained in 2004 and 2010 (Popov et al. 2005, 2011), respectively, showing normal hearing thresholds and suggesting minimal impact of underwater noise on their hearing by the time of testing. However, this does not necessarily imply that aquarium noise will never affect the finless porpoises. Many infrastructure construction projects involving pile driving, which generates intense noise, have been carried out in the immediate area of the Baiji Aquarium since 2010. However, due to the absence of systematic measurements of underwater noise in the Baiji Aquarium, we cannot evaluate the noise exposure history for the finless porpoises after 2010. Testing the audiograms of the same animals previously examined or other contemporary finless porpoises in the Baiji Aquarium that are experiencing similar noise conditions as Taotao may provide partial insights into this issue and warrants further investigation.

The noise‐induced threshold shift in partial frequencies of a harbor seal recovered 1 year after the exposure event (Reichmuth et al. 2019a). We cannot exclude the possibility that Taotao's audiogram, or at least specific frequency bands within it, may reflect a TTS that could return to normal under quiet conditions, warranting further investigation.

Attention should be drawn to the fact that compared to the slope‐intercept method for threshold determination and the root mean square (rms) sound pressure level across the entire pip train used to calibrate the stimulus signals in previous finless porpoise hearing research (Wang et al. 2020b), both the statistical response detection of the AEP threshold and the peSPL used to calibrate the stimulus signals in this study may result in higher thresholds (Houser et al. 2025).

### 4.2 Noise in Aquarium

The persistent neglect of anthropogenic noise pollution in aquariums and its impact on aquarium animals has been evident (Heise et al. 2016; O'neal 1998; Wang et al. 2025). The mean 1/3‐octave band power spectra from 500 Hz to 50 kHz at the Baiji Aquarium (ranging from 78 to 88 dB) were lower than those recorded at the Vancouver Aquarium during routine operations, which ranged from 87.0 to 104.5 dB across the same frequency range (Heise et al. 2016). The underwater noise levels in the Baiji Aquarium were lower than those measured in the Monterey Bay Aquarium under standard exhibit conditions. Specifically, within the broadband frequency range of 0–6.4 kHz, noise levels in the Monterey Bay Aquarium ranged from 112 to 125 dB re 1 µPa (O'neal 1998). It is evident that underwater sound levels in the aquarium setting are often elevated. Given the impulsive nature of conspecific‐generated sounds, whose impacts are frequently underestimated, coupled with the observation that underwater sound exposure levels at the Baiji Aquarium surpass the PTS onset threshold for impulsive noise in finless porpoises, it underscores the critical necessity to mitigate underwater noise pollution and its potential adverse effects on aquatic animals in captivity. Additionally, it is possible that sounds from conspecifics have partially contributed to the notches observed in Taotao's audiograms.

The underwater noise below 1 kHz in the Baiji Aquarium may be attributed to the life support pump systems (see Figure 3). The noise above 90 kHz, reaching a peak at 125.9 kHz, was mainly attributed to the biosonars of the porpoises. This conclusion is supported by the average peak frequency of the echolocation signal emitted by Yangtze finless porpoises in the aquarium, which was recorded at 134 ± 10 kHz (mean ± SD), with a range spanning from 118 to 149 kHz (Fang et al. 2015). In this study, no clear direct correlation was observed between the notches at 20–40 kHz and 90 kHz in Taotao's audiogram and the power spectra of the ambient sound levels measured at the Baiji Aquarium. This could be partly due to the relatively short sampling duration (spanning 7 days) of ambient noise in the Baiji Aquarium in this study. Furthermore, we have no indication of any noisy activities occurring between the time the ambient sound levels were recorded and the porpoise auditory test was conducted. While the hearing threshold of very high‐frequency hearing group cetaceans to low‐frequency sound is generally high, cetaceans may possess the ability to perceive vibrotactile signals at frequencies below 1 kHz (Au 1993). Additionally, exposure to low‐frequency pitches, even if inaudible to the animal, can still alter the functioning of the inner ear, making it temporarily more prone to damage after such exposure (Williams 2014). Therefore, low‐frequency sound remains a concern for them.

### 4.3 Broader Significance and Further Study

While the survival rate of marine mammals in zoological institutions has substantially improved (Tidière et al. 2023), the elevated noise exposure due to prolonged opening hours of Nanning Aquarium during tourist rush seasons has previously been observed to result in TTSs exceeding 20 dB in 41% of Indo‐Pacific humpback dolphin audiograms (Wang et al. 2025). However, the hearing thresholds returned to their normal range when the opening hours were reduced during the tourist off‐seasons (Wang et al. 2025). In this study, the audiogram of the world's first successfully captive‐born and bred Yangtze finless porpoise demonstrated threshold levels more than 40 dB higher than previously published data for finless porpoise audiograms. Since the underwater noise in the Baiji Aquarium surpasses the TTS onset threshold for finless porpoises, it is crucial to prioritize welfare concerns regarding the impact of underwater sound from conspecifics on captive animals. Ensuring optimal underwater sound conditions is crucial for the welfare of marine mammals in captivity. The routine incorporation of underwater sound monitoring should be integrated as a standard component in water quality assessment protocols for facilities housing species with heightened sensitivity to acoustic stimuli. Management and mitigation strategies aimed at reducing the impact of elevated aquarium noise on porpoises involve optimizing and controlling the noise generated by the water circulation system within the aquarium. This may include measures such as increasing the distance between the pump and the engine of the water circulation system or installing sound isolation barriers between them. Porpoises are highly vocal animals, with the Yangtze finless porpoise observed emitting intense echolocation click trains, reaching over 148 dB re 1 µPa peak‐to‐peak, approximately every 6.4 m in the wild (Akamatsu et al. 2007). Due to the limited space in aquarium settings, cetaceans may not have sufficient room to escape the echolocation soundscapes emitted by other individuals. Therefore, the adequacy of aquarium space and animal group size should be thoroughly evaluated. In an aquarium setting, the navigation and prey capture functions of cetaceans' echolocation signals are of lesser importance. Therefore, consideration should be given to the incorporation of sound absorption materials or structures designed to absorb echolocation signals and reduce reverberation properties, particularly in concrete pool settings, during the aquarium construction process. Furthermore, infrastructure construction that produces intense sounds, whether within the aquarium or in nearby areas should also be taken into consideration.

The underwater noise in the Baiji Aquarium comprised both non‐impulsive and impulsive sounds due to the high production rate of echolocation signals by the finless porpoise (Figure 4), which became integrated with the ambient noise. Presently, threshold standards exist for marine mammals' temporary or PTS onset when exposed to either non‐impulsive or impulsive noise individually (Southall et al. 2019). Kurtosis, a measure of the asymmetry in the probability distribution of acoustic pressures, has been proposed as a method to differentiate between impulsive and non‐impulsive underwater anthropogenic sounds (Martin et al. 2020). It has been suggested as a parameter for predicting hearing loss in marine mammals from exposure to sounds with varying amounts of impulsiveness (Guan and Brookens 2023; Guan et al. 2022; Zeddies et al. 2023). However, criteria for noise exposure in marine mammals exposed to complex sounds, comprising both non‐impulsive and impulsive characteristics, have not yet been established and warrant further investigation.

To facilitate comparison with previous hearing studies on the finless porpoise, the same audiogram measurement using stimuli with a constant tone pip duration was employed. However, this type of stimulus may not be fully suitable for low frequencies, as the lower the carrier frequency, the fewer cycles are present in the pip, resulting in a broader signal spectrum. Future studies should consider using stimuli with a constant number of cycles and varying pip durations.

Whether captive‐born odontocetes partially lose high‐frequency sensitivity deserves further analysis. As we now have more animals born in captivity, including two of Taotao's offspring, it would be vital to examine the hearing of Taotao's offspring in the future. Further investigation into systematic underwater noise monitoring and its impact on the finless porpoise in the Baiji Aquarium is warranted.

## 5 Conclusion

Aquarium noise and its potential impact on the hearing of cetaceans were analyzed through a combined assessment of underwater noise levels in the Baiji aquarium and the hearing thresholds of a Yangtze finless porpoise that was the world's first successful birth and survival of a finless porpoise in captivity. The results indicate that the audiogram of the finless porpoise exhibited a 40 dB elevation in the threshold compared to the previously published audiograms of the Yangtze finless porpoise. Both a congenital hearing disorder and elevated sound exposure levels from conspecifics in the Baiji Aquarium could have contributed to the hearing loss in Taotao. Our results highlight the need to consider the impacts of conspecifics sound on cetaceans housed in aquariums and have the potential to enhance comprehension of the welfare of marine mammals in captivity.

## Ethics Statement

This research adhered to ethical guidelines and regulations concerning the ethical use of animals in experiments, as outlined in the Law of the People's Republic of China on the Protection of Wildlife. Approval for all research activities was obtained from the Animal Care and Use Committees of the Institute of Hydrobiology of the Chinese Academy of Sciences, as well as from the Ministry of Agriculture and Rural Affairs of China (Permit numbers: E0550104 and Y81Z161).

## Conflicts of Interest

The authors declare no conflicts of interest.

## Supporting information




**Figure S1** (A) Rhythmic tone pips with the pip train stimulus administered at a rate of 10 cycles per second (Only the first 200 ms signal slice of the rhythmic tone pips was presented here). (B) Stimulus consisted of 20 individual pips. (C) each pip signal lasting 0.25 milliseconds and subjected to cosine‐envelope modulation. Fig. S1B was expanded from Fig. S1A over the time span of 0–20 ms, while Fig. S1C was expanded from Fig. S1B over the time span of 0–1 ms.

## Data Availability

The data that support the findings of this study are available from the corresponding author upon reasonable request.
