# Supplementary material for: Severe Hearing Loss in the World's First Successfully Captive‐Born Yangtze Finless Porpoise: Impact of High Underwater Sound Exposure and Congenital Hearing Disorders
Source: Integr Zool. 2025 Apr 6;21(2):331–42. doi: 10.1111/1749-4877.12973 (PMC12971628; doi:10.1111/1749-4877.12973)
Supplement: Supplementary file 1 — Figure S1 (A) Rhythmic tone pips with the pip train stimulus administered at a rate of 10 cycles per second (Only the first 200 ms signal slice of the rhythmic tone pips was presented here). (B) Stimulus consisted of 20 individual pips. (C) each pip signal lasting 0.25 milliseconds and subjected to cosine‐envelope modulation. Fig. S1B was expanded from Fig. S1A over the time span of 0–20 ms, while Fig. S1C was expanded from Fig. S1B over the time span of 0–1 ms. [file INZ2-21-331-s001.docx]

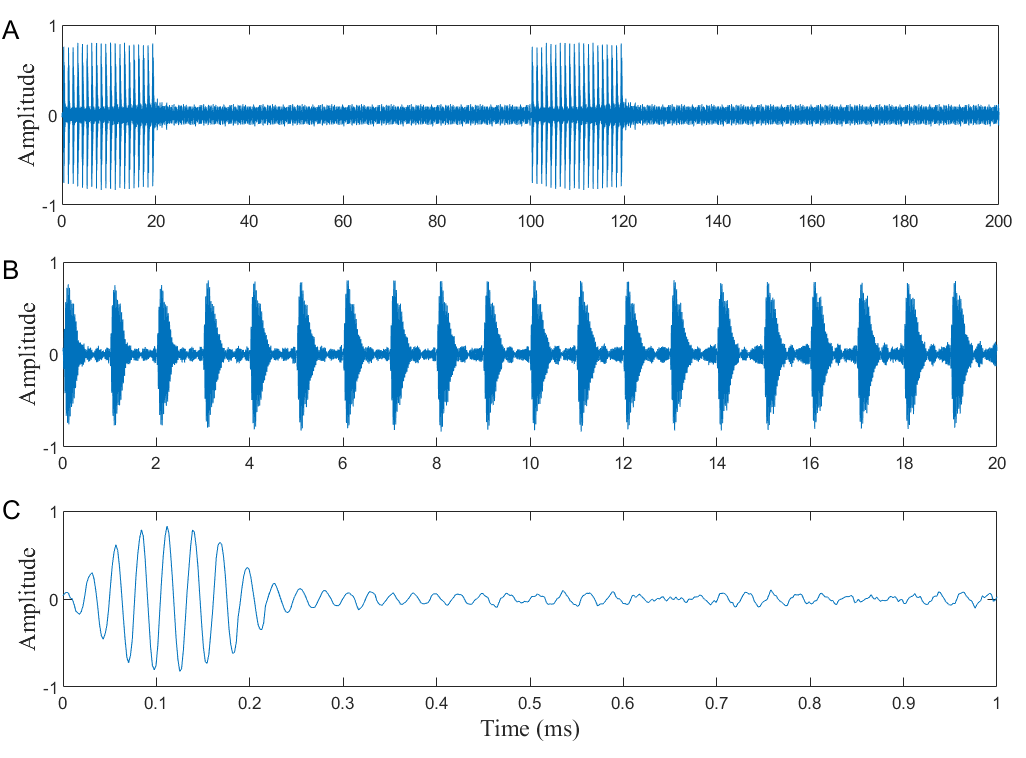


**Figure S1** (A) Rhythmic tone pips with the pip train stimulus administered at a rate of 10 cycles per second (Only the first 200 ms signal slice of the rhythmic tone pips was presented here). (B) Stimulus consisted of 20 individual pips. (C) each pip signal lasting 0.25 milliseconds and subjected to cosine-envelope modulation. FIG. B was expanded from FIG. A over the time span of 0-20 ms, while FIG. C was expanded from FIG. B over the time span of 0-1 ms.
